# Univariate and multivariate analyses of preoperative factors influencing symptomatic outcomes of transoral fundoplication

**DOI:** 10.1007/s00464-014-3557-z

**Published:** 2014-05-31

**Authors:** Reginald C. W. Bell, Mark A. Fox, William E. Barnes, Peter G. Mavrelis, Robert W. Sewell, Bart J. Carter, Glenn M. Ihde, Karim S. Trad, David Dargis, Kevin M. Hoddinott, Katherine D. Freeman, Tanja Gunsberger, Mark G. Hausmann, Brian DaCosta Gill, Erik Wilson

**Affiliations:** 1 SurgOne Foregut Institute, 401 West Hampden Place, Suite 230, Englewood, CO 80110 USA; 2 Crossville Medical Group, PA, Crossville, TN USA; 3 Livingston Hospital and Healthcare Services, Inc. CAH, Salem, KY USA; 4 Internal Medicine Associates, Merrillville, IN USA; 5 Master Center for Minimally Invasive Surgery, Southlake, TX USA; 6 Mt. Graham Regional Medical Center, Safford, AZ USA; 7 Ihde Surgical Group, Arlington, TX USA; 8 The George Washington University School of Medicine and Health Sciences, Washington, DC USA; 9 Reston Surgical Associates, Reston, VA USA; 10 Allegan Surgical Associates, Allegan, MI USA; 11 Munroe Regional Medical Center, Ocala, FL USA; 12 Tempe St. Luke’s Hospital, Tempe, AZ USA; 13 The Surgeons Group of Baton Rouge, Baton Rouge, LA USA; 14 Utah County Surgical Associates, Provo, UT USA; 15 University of Texas Health Science Center, Houston, TX USA

**Keywords:** Heartburn, EsophyX, Gastroesophageal reflux, TIF, Regurgitation, GERD, Refractory

## Abstract

**Background:**

Preoperative factors predicting symptomatic improvement after transoral fundoplication (TF) in chronic gastroesophageal reflux disease (GERD) patients with persistent symptoms on proton-pump inhibitors (PPIs) therapy have not been elucidated fully.

**Methods:**

Univariate and multivariate logistic regression analyses were performed on data from 158 consecutive patients who underwent TF with the EsophyX device between January 2010 and June 2012 in 14 community centers. Variables included age, gender, body mass index, GERD duration, PPIs therapy duration, presence of hiatal hernia, esophagitis, Hill grade, quality of life scores (QOL) on PPIs, % total time pH < 4, and DeMeester score on reflux testing off PPIs.

**Results:**

All patients suffered from typical GERD symptoms. Additionally, 78 % (124/158) of patients suffered from atypical symptoms. Six percent (10/158) with recurrent GERD symptoms refractory to PPI therapy underwent revisional procedure (9 laparoscopic Nissen, 1 TF). Median follow-up was 22 (range 10–43) months. For patients with typical symptoms, univariate analyses revealed 4 preoperative factors predictive of successful outcomes: age ≥ 50 [odds ratio (OR) = 2.4, 95 % confidence interval (CI) = 1.2–4.8, *p* = 0.014], GERD Health-related Quality of Life score (GERD-HRQL) ≥ 15 on PPIs (OR = 6.0, CI = 1.2–29.4, *p* = 0.026, Reflux Symptom Index score  > 13 on PPIs (OR = 2.4, CI = 1.1–5.2, *p* = 0.027), and Gastroesophageal Reflux Symptom Score  ≥ 18 on PPIs (OR = 2.6, CI = 1.2–5.8, *p* = 0.018). Age and GERD-HRQL score remained significant predictors by multivariate analysis. For patients with atypical symptoms, only GERD-HRQL score ≥ 15 on PPIs (OR = 9.9, CI = 0.9–4.6, *p* = 0.036) was associated with successful outcomes.

**Conclusions:**

Elevated preoperative QOL scores on PPIs and age ≥ 50 were most closely associated with successful outcome of TF in patients with persistent symptoms despite medical therapy.

The successful management of chronic gastroesophageal reflux disease (GERD) includes controlling troublesome reflux symptoms, improving patients’ quality of life (QOL), and preventing complications [1, 2]. Despite the prevalent use of lifestyle modification, high-dose proton-pump inhibitor (PPI) therapy and other medications, 20–40 % of medically treated GERD patients continue to have persistent troublesome symptoms [3]. Although treatment of GERD with laparoscopic fundoplication (LF), when performed in specialty centers, historically reports an excellent relief of typical GERD symptoms [4], the procedure is performed in a minor percentage of patients with persistent symptoms despite medical therapy and nationally this number is decreasing. Presence of typical symptoms and good response to PPI therapy have predicted success with LF treatment [5]. Partly with the goal of providing better outcomes to these patients with persistent symptoms despite medical therapy, endoscopic antireflux procedures have been evaluated as potential alternatives to those who do not wish to have LF. This study evaluated factors that might be predictive of successful symptomatic results after one such endoscopic antireflux procedure.

Transoral fundoplication (TF) using the EsophyX device has been described previously [6–10]. Under general anesthesia, the device is introduced over a flexible endoscope into the esophagus and stomach. A tissue mold at the end of the device apposes the gastric fundus to the distal esophagus, and small (6–7 mm) polypropylene H-shaped fasteners are placed from esophageal to fundic lumen to create the plication. An endoluminal esophagogastric fundoplication can be created up to 4 cm in length and 270° circumference as confirmed by endoscopic evaluation.

Although some reports of TF evaluated variables associated with clinical outcomes as a portion of the report [6, 11–13], to date there is no single study that primarily investigated association of preoperative factors with symptomatic outcomes of TF. In this study, we evaluated multiple preoperative factors and their relationship to clinical outcomes of TF in GERD patients with an inadequate response to PPIs.

## Patients and methods

### Study design

A prospective 14-center community practice registry was established in 2010 (Clinicaltrials.gov identifier NCT01118585). The primary endpoint of the study was elimination of troublesome GERD symptoms in patients undergoing TF using the EsophyX device. None of the patients in the study had concomitant crural closure. Outcomes were evaluated using disease-specific questionnaires. The current study evaluated patient variables with regard to the success of the procedure.

The following preoperative measures were recorded: age, gender, body mass index (BMI), GERD duration, PPI therapy duration, presence of hiatal hernia, Los Angeles (LA) classification of esophagitis, Hill grade of the gastroesophageal (GE) valve, and GERD-specific QOL scores on PPIs. The QOL questionnaires administered were the GERD Health-related Quality of Life (GERD-HRQL) score, Reflux Symptom Index (RSI) score, and Gastroesophageal Reflux Symptom Score (GERSS).

### Study variables

Variables were divided into binary groups for analysis as follow: gender (male or female), age (<50 or ≥50 years old), GERD duration (<5 or ≥5 years), duration of PPI use (<5 or ≥5 years), BMI (<30 or ≥30), hiatal hernia (present or absent), esophagitis (present or absent), GERD-HRQL < 15 or ≥15, RSI ≤ 13 or >13, GERSS **<** 18 or ≥18. Use of proton-pump inhibitors were categorized as daily, occasional, or none.

Outcomes were assessed using QOL questionnaires and recording of acid-suppressive medication use at defined postoperative time periods (6, 12, 24, and 36 months).

### Patient population

The study population consisted of 158 patients enrolled in the multicenter registry who underwent TF with EsophyX device without crural closure. The procedures were performed in 14 community-based centers across the United States between January 2010 and June 2012 following the previously described 2.0 protocol [14]. Primary inclusion criteria were (1) GERD symptoms for at least 1 year, (2) history of daily PPI use for at least 6 months even if patients were not currently taking PPIs, (3) hiatal hernia ≤2 cm in axial length and ≤3 cm in greatest transverse dimension, and (4) willingness to provide informed consent. Primary exclusion criteria were (1) a BMI greater than 35 kg/m^2^, (2) esophagitis grade D (LA classification), (3) GE junction classified as Hill grade IV, (4) advanced disease including long segment Barrett’s esophagus, esophageal ulcer, and fixed esophageal stricture or narrowing. All patients in this study had typical GERD symptoms and objective documentation of GERD.

### Preoperative evaluation

Preoperative evaluation included symptom assessment using GERD-HRQL, RSI, and GERSS questionnaires. Objective documentation of GERD was obtained from upper endoscopy findings of esophagitis, non-fixed stricture, columnar lined epithelium with Barrett esophagus on biopsy (limited to ≤2 cm by study protocol), and/or abnormal ambulatory reflux testing.

### Intervention and postoperative care

Transoral esophagogastric endoscopic fundoplication using the EsophyX_2_ device (EndoGastric Solutions, Inc, Redmond, Washington) without crural repair was performed using a defined protocol (2.0). The main goal of the procedure was to create a GE valve 270° or more in circumference and 2–5 cm in length. This was achieved by endoluminal folding of the fundus of the stomach around the distal esophagus and securing it in place with multiple (12–30) “H”-shaped polypropylene fasteners. Detailed technical aspects of the procedure have been previously described [14].

Following the procedure, patients were asked to continue PPI therapy for 2 weeks and to adhere to a modified diet for 4–6 weeks. Anti-emetic prophylaxis was routinely administered in the first 24 h after the procedure and then as needed. Patients were asked to desist from rigorous physical activities for 4–6 weeks after the procedure.

### Outcome measures

Three validated self-reported disease-specific questionnaires evaluated symptom severity prior to and after the procedure. The GERD-HRQL evaluates heartburn (six questions), dysphagia (two), bloating (one), and the impact of medication on daily life (one) on a scale from 0 (no symptoms) to 5 (incapacitating symptoms). The scale combines frequency and severity of symptoms in a single response. Total score on the GERD-HRQL ranges from 0 to 50, with higher scores indicating more severe GERD [15, 16]. There is not a defined “normal” score; instead a 50 % improvement in total GERD-HRQL score is indicative of a successful therapeutic outcome. RSI measures symptoms associated with extraesophageal manifestations of GERD (atypical symptoms) [17]. Each of nine symptom scores can range from 0 (no symptoms) to 5 (severe symptoms), with a maximum total score of 45. A total RSI score of ≤13 is considered normal [17]. The GERSS questionnaire assesses classic (heartburn and regurgitation) and atypical GERD symptoms (abdominal distension, dysphagia, and cough) [18, 19]. Specific symptom items are scored as a product of severity (0 = no symptoms at all to 3 = severe symptoms) and frequency (0 = never to 4 = daily). These five symptom scores (range 0–12) are then summed to create the total GERSS score (range 0–60). A total GERSS < 18 indicates controlled reflux symptoms and is considered normal [19]. All patients completed preoperative questionnaires on PPIs. At follow-up, patients completed QOL questionnaires without altering their current GERD medical treatment, if any.

PPI usage was defined as “none” (medication not taken at all), “occasional” (if any dose was taken ≤3 days a week), or “daily” (if any dose was taken >3 days a week).

### Definition of symptomatic treatment outcomes: successful, responsive, poor

Clinical outcome was considered *successful* if patients experienced ≥50 % reduction of a GERD-HRQL score or normalization of RSI score (≤13) and were completely off PPIs. Outcome was considered *responsive* if patients experienced ≥50 % reduction in GERD-HRQL score or normalization of RSI regardless of ongoing medication use (as long as the dosing of medication did not increase). An outcome was considered *poor* for patients who experienced a <50 % reduction in GERD-HRQL or had abnormal RSI at follow-up, or underwent reoperation or increased their medication use after surgery. Analyses were performed separately on patients with typical and atypical symptoms before TF.

### Data collection and statistical analyses

All data were prospectively collected. Descriptive statistics were calculated for variables of interest. Univariate and multivariate logistic regression analyses were performed to identify factors predictive of successful symptomatic outcomes following TF. Covariate with Wald’s *p* values ≤ 0.25 on univariate analyses was entered into backwards stepwise multivariate regression analyses. *p* value ≤ 0.05 was considered significant. Descriptive results are reported as median (range). Categorical data were reported as proportions and counts. Individual non-parametric Wilcoxon signed rank tests were performed to compare improvement in QOL scores at 22-month follow-up versus baseline. McNemar’s test was performed to compare proportions of paired data. A *p* value < 0.05 was considered to be statistically significant for these tests. All analyses were performed using JMP 10.0 statistical program.

## Results

### Patient characteristics

**Fig. 1:**
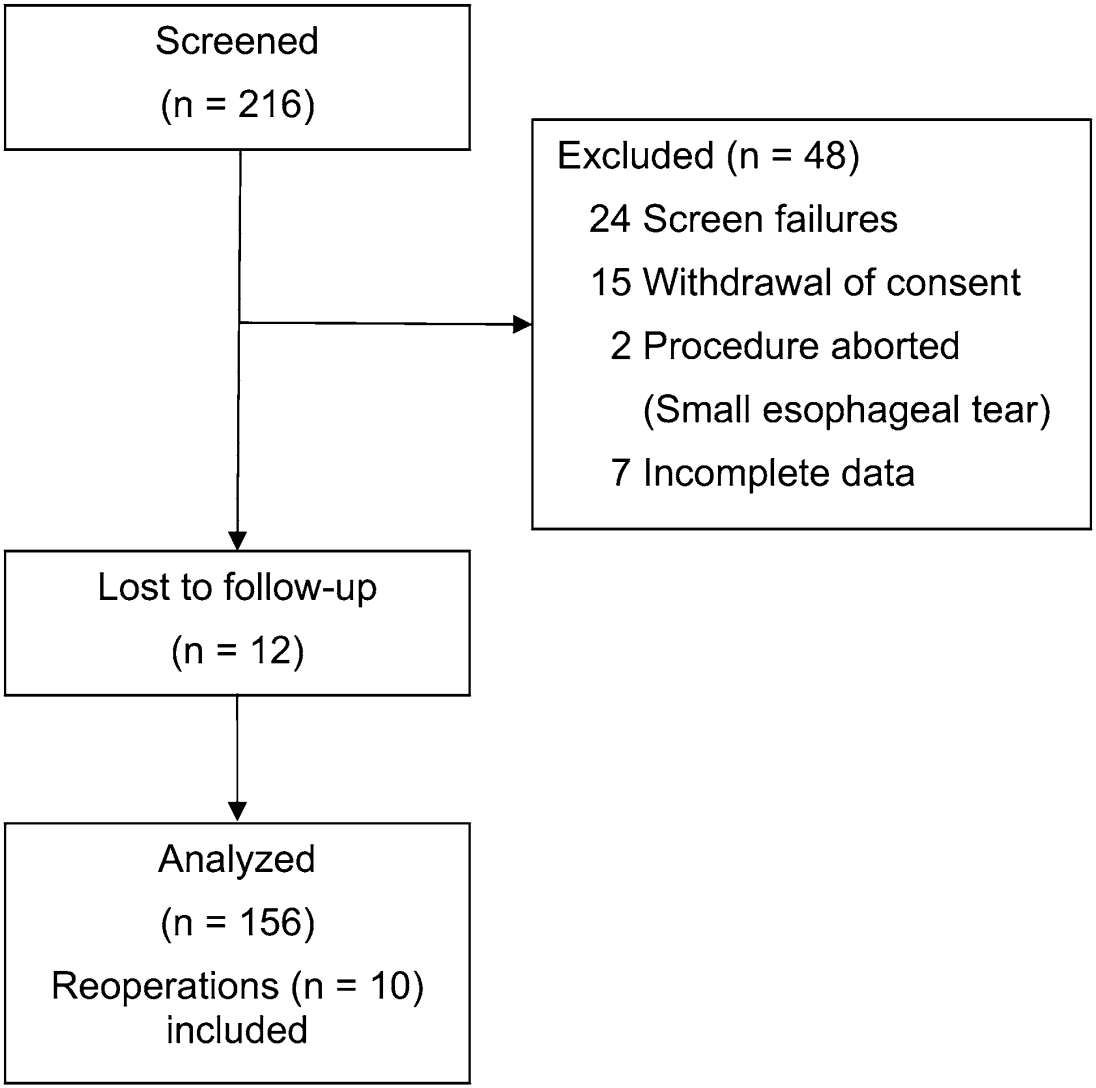
Flowchart of patients enrolled in the study

This study enrolled 158 patients with medically refractory typical GERD symptoms who underwent TF without crural repair between January 2010 and June 2012 (Fig. 1). Median follow-up was 22 (range 10–43) months. The median age was 59 years (range 19–90 years), and 29 % of patients were male. The study population included 10 patients (6 % of study population) who developed recurrent GERD symptoms after TF and decided to undergo a revisional procedure (9 laparoscopic Nissen fundoplication and 1 TF). For QOL comparisons, these patients were considered to have poor outcomes, and preoperative QOL scores were imputed for follow-up visits. Demographics and baseline characteristics of study patients are summarized in Table 1.

**Table 1:** Baseline characteristics of study patients

Characteristics	Frequency (*n* = 158)
Gender	
Male, *n* (%)	46 (29)
Female, *n*(%)	112 (71)
Age, median (range)	58.5 (19–90)
<50, *n* (%)	47 (30)
≥50, *n* (%)	111 (70)
Body mass index, kg/m^2^	
<30, *n* (%)	117 (74)
≥30, *n* (%)	41 (26)
Esophagitis (Los Angeles Grade), *n* (%)	107 (68)
A, *n* (%)	30 (28)
B, *n* (%)	63 (59)
C, *n* (%)	14 (13)
Hiatal hernia	
Yes, *n* (%)	113 (72)
No, *n* (%)	45 (28)
GERD duration^a^	
<5, *n* (%)	34 (23)
≥5, *n* (%)	110 (77)
PPI duration^a^	
<5, *n* (%)	52 (36)
≥5, *n* (%)	92 (64)
GERD-HRQL score on PPI therapy	
<15, *n* (%)	10 (6)
≥15, *n* (%)	148 (94)
RSI score on PPI therapy^b^	
≤13, *n* (%)	34 (22)
>13, *n* (%)	123 (78)
GERSS score on PPI therapy^c^	
<18, *n* (%)	32 (21)
≥18, *n* (%)	120 (79)
% total time pH < 4^d^	
≥8 %, *n*/*n* (%)	13/27 (48)
<8 %, *n*/*n* (%)	14/27 (52)
Total DeMeester score on 48-h testing^e^	
≥30, *n*/*n* (%)	7/23 (30)
<30, *n*/*n* (%)	16/23 (70)

*GERD* gastroesophageal reflux disease, *GERD-HRQL* gastroesophageal reflux disease health-related quality of life, *RSI* reflux symptom index, *GERSS* gastroesophageal reflux symptom score, *PPI* proton-pump inhibitor

^a^Data were available for 144 patients

^b^One patient with incomplete questionnaire was excluded form analyses

^c^Six patients with incomplete questionnaires were excluded from analyses

^d^Data were available for 27 patients

^e^Data were available for 23 patients

In addition to having typical GERD symptoms, 78 % (124/158) of patients suffered from atypical symptoms (RSI score > 13). An additional analysis was conducted looking for predictive factors for outcomes of atypical symptoms in these patients.

### Patients with typical GERD symptoms

All 158 patients had preoperative troublesome typical GERD symptoms on PPIs, defined as having at least one of the individual items on GERD-HRQL scored > 2. Additionally, all 158 patients had a total GERD-HRQL ≥ 10 on PPIs at entry. The median GERD-HRQL score improved from 28 (10–50) on PPIs to 5 (0–45) at follow-up, *p* < 0.001. 75 % of patients (118/158) experienced a ≥50 % reduction in their GERD-HRQL score. All patients were on PPIs at screening. Complete cessation of PPI therapy occurred in 70 % (110/158) of patients; an additional 7 % (11/158) of patients were only taking PPIs occasionally. 23 % (37/158) of patients remained on daily PPI therapy. No patient had increased their PPI dose at follow-up as compared with baseline. A responsive outcome was achieved in 75 % (119/158) of patients (≥50 % reduction of GERD-HRQL regardless of continuing medication use). A successful outcome was achieved in 58 % (91/158) of patients (≥50 % reduction of GERD-HRQL and no PPI use). Outcomes were considered poor in 25 % (39/158) of patients with typical symptoms.

Predictive factors of a *successful* outcome (off PPIs and ≥50 % improvement in the total GERD-HRQL score) by univariate analysis were (1) age ≥ 50 [odds ratio (OR) = 2.4, 95 % confidence interval (CI) = 1.2–4.9, *p* = 0.014], (2) preoperative GERD-HRQL score ≥ 15 on PPIs (OR = 6.0, CI = 1.5–40.1, *p* = 0.026), (3) preoperative RSI score > 13 on PPIs (OR = 2.4, CI = 1.1–5.3, *p* = 0.027), and (4) preoperative GERSS score ≥ 18 on PPIs (OR = 2.6, CI = 1.2–5.9, *p* = 0.018). Female gender (OR = 2.0, CI = 1.0–4.0, *p* = 0.053) and presence of esophagitis on endoscopy (OR = 1.9, CI = 1.0–3.7, *p* = 0.065) closely approached significant level (Table 2). By multivariate analysis, age ≥ 50 (OR = 2.6, CI = 1.3–5.3, *p* = 0.008) and initial GERD-HRQL score ≥ 15 on PPIs (OR = 7.1, CI = 1.7–47.5, *p* = 0.017) were predictive of a *successful* outcome (Table 3). When age was ≥50 and GERD-HRQL score was ≥15 *on* PPIs preoperatively, a *successful* outcome was achieved in 67 % (68/102) of patients with inadequate control of symptoms despite medical therapy. Inversely, if both factors were unfavorable (age < 50 and preoperative GERD-HRQL score on PPIs < 15), 20 % (3/15) of patients achieved a *successful* outcome.

**Table 2:** Univariate regression analyses for successful resolution of classic GERD symptoms

Variable	Successful outcome	Unadjusted OR (95 % CI)	*p* value^a^
Age (years)			
<50	20/47 (43)		
≥50	71/111 (64)	2.4 (1.2–4.9)	0.014
Gender			
Male	21/46 (46)		
Female	70/112 (63)	2.0 (1.0–4.0)	0.053
Body mass index (kg/m^2^)			
<30	67/117 (57)		
≥30	24/41 (59)	1.1 (0.5–2.2)	0.887
Hiatal hernia			
No	22/45 (49)		
Yes	69/113 (61)	1.6 (0.8–3.3)	0.164
Esophagitis			
No	24/51 (47)		
Yes	67/107 (63)	1.9 (1.0–3.7)	0.065
GERD duration (years)			
<5	20/34 (59)		
≥5	63/112 (56)	0.9 (0.4–2.0)	0.862
PPI duration (years)			
<5	31/52 (60)		
≥5	51/92 (55)	0.8 (0.4–1.7)	0.627
GERD-HRQL score on PPIs			
<15	2/10 (20)		
≥15	89/148 (60)	6.0 (1.5–40.1)	0.026
RSI score on PPIs			
≤13	14/34 (41)		
>13	77/123 (63)	2.4 (1.1–5.3)	0.027
GERSS score on PPIs			
<18	13/32 (41)		
≥18	77/120 (64)	2.6 (1.2–5.9)	0.018
% total time pH < 4			
≥8 %	4/13 (31)		
<8 %	7/14 (50)	2.3 (0.5–11.7)	0.313
Total DeMeester score			
≥30	2/7 (29)		
<30	8/16 (50)	1.8 (0.3–10. 4)	0.539

Data are expressed n/n (%)

*GERD* gastroesophageal reflux disease, *GERD-HRQL* gastroesophageal reflux disease health-related quality of life, *GERSS* gastroesophageal reflux symptom score, *OR* odds ratio, *PPIs* proton-pump inhibitors, *RSI* reflux symptom index

^a^Wald *p* value

**Table 3:** Multivariate backward stepwise regression analysis for successful resolution of classic GERD symptoms

Variable	Successful outcome		Successful or responsive outcome	
	OR (95 % CI)	*p* value	OR (95 % CI)	*p* value
Age (years)				
<50	–	–	–	–
≥50	2.6 (1.3–5.3)	0.008	2.6 (1.2–5.7)	0.018
GERD-HRQL score on PPIs				
<15	–	–	–	–
≥15	7.1 (1.7–48.5)	0.017	–	–
Esophagitis				
No	–	–	–	–
Yes	–	–	2.9 (1.3–6.3)	0.008

*GERD-HRQL* gastroesophageal reflux disease health-related quality of life, *OR* odds ratio, *PPIs* proton-pump inhibitors

Using univariate analysis, factors identified as predicting a *responsive* outcome from TF for patients with typical GERD symptoms were the presence of esophagitis on endoscopy (OR = 2.6, CI = 1.2–5.5, *p* = 0.013), age ≥ 50 years (OR = 2.3, CI = 1.1–4.9, *p* = 0.032), and GERSS > 18 on PPIs (OR = 2.4, CI = 1.0–5.6, *p* = 0.042). Female gender and a GERD-HRQL ≥ 15 *on* PPIs approached statistical significance (Table 4). Preoperative factors predictive of *responsive* outcome of typical GERD symptoms to TF by multivariate analysis were age ≥ 50 years (OR = 2.6, CI = 1.2–5.7, *p* = 0.018) and the presence of esophagitis (OR = 2.9, CI = 1.3–6.3, *p* = 0.008), Table 3. When both predictive factors were favorable, 84 % (61/73) of patients achieved a *responsive* outcome of typical GERD symptoms. If both factors were unfavorable, 30 % (3/10) of patients had a *responsive* outcome.

**Table 4:** Univariate regression analyses for successful or responsive resolution of classic GERD symptoms

Variable	Successful or responsive outcome	Unadjusted OR (95 % CI)	*p* value^a^
Age (years)			
<50	30/47 (64)		
≥50	89/111 (80)	2.3 (1.1–4.9)	0.032
Gender			
Male	30/46 (65)		
Female	89/112 (80)	2.1 (1.0–4.4)	0.062
Body mass index (kg/m^2^)			
<30	88/117 (75)		
≥30	31/41 (76)	1.0 (0.5–2.4)	0.960
Hiatal hernia			
No	31/45 (69)		
Yes	88/113 (78)	1.6 (0.7–3.4)	0.239
Esophagitis			
No	32/51 (63)		
Yes	87/107 (81)	2.6 (1.2–5.5)	0.013
GERD duration			
<5	27/34 (79)		
≥5	84/112 (75)	0.8 (0.3–1.9)	0.598
PPI duration			
<5	38/52 (73)		
≥5	71/92 (77)	1.2 (0.6–2.7)	0.582
GERD-HRQL score on PPIs			
<15	5/10 (50)		
≥15	114/148 (77)	3.4 (0.9–12.7)	0.068
RSI score on PPIs			
≤13	22/34 (65)		
>13	96/123 (78)	1.9 (0.8–4.4)	0.115
GERSS score on PPIs			
<18	20/32 (63)		
≥18	96/120 (80)	2.4 (1.0–5.6)	0.042
% total time pH < 4			
≥8 %	6/13 (46)		
<8 %	8/14 (57)	1.6 (0.3–7.4)	0.569
Total DeMeester score			
≥30	3/7 (43)		
<30	9/16 (56)	1.3 (0.3–6.4)	0.736

Data are expressed n/n (%)

*GERD* gastroesophageal reflux disease, *GERD-HRQL* gastroesophageal reflux disease health-related quality of life, *GERSS* gastroesophageal reflux symptom score, *OR* odds ratio, *PPIs* proton-pump inhibitors, *RSI* reflux symptom index

^a^Wald *p* value

### Atypical GERD symptoms

Of the 158 patient cohort, 78 % (124/158) also had atypical symptoms and an RSI score on PPIs of >13 at presentation. In this subgroup, the median RSI score improved from 26 (14–45) to 5.5 (0–41), *p* < 0.001. 74 % (91/124) of patients were completely off PPIs, and an additional 6 % (7/124) of patients were on occasional PPI therapy. 21 % (26/124) of patients remained on daily PPI therapy. Median follow-up was 22 (10–43) months.

Among these patients with atypical GERD symptoms, a *successful* outcome (off PPIs and the total RSI score ≤ 13) was observed in 60 % (74/124) of patients. A *responsive* outcome (on any dose PPIs and RSI score ≤ 13) was observed in 75 % (93/124) of patients. Outcomes were considered *poor* in 25 % (31/124) of patients with atypical symptoms.

The only predictor of a *successful * outcome of atypical GERD symptoms by univariate analysis was a total preoperative GERD-HRQL score ≥ 15 on PPIs (OR = 10.0, CI = 1.6–191.2, *p* = 0.036). The presence of esophagitis approached statistical significance (Table 5).

**Table 5:** Univariate regression analyses for successful resolution of atypical GERD symptoms

Variable	Successful outcome	Unadjusted OR (95 % CI)	*p* value^a^
Age (years)			
<50	20/36 (56)		
≥50	54/88 (61)	1.3 (0.6–2.8)	0.550
Gender			
Male	15/30 (50)		
Female	59/94 (63)	1.7 (0.7–3.9)	0.217
Body mass index (kg/m^2^)			
<30	52/92 (57)		
≥30	22/32 (59)	1.7 (0.7–4.1)	0.227
Hiatal hernia			
No	17/35 (49)		
Yes	57/89 (64)	1.9 (0.9–4.2)	0.116
Esophagitis			
No	15/32 (47)		
Yes	59/92 (64)	2.0 (0.9–4.6)	0.089
GERD duration			
<5	17/27 (63)		
≥5	52/87 (604)	0.9 (0.3–2.1)	0.767
PPI duration			
<5	28/42 (67)		
≥5	41/71 (58)	0.7 (0.3–1.5)	0.348
GERD-HRQL score on PPIs			
<15	1/7 (14)		
≥15	73/117 (62)	10.0 (1.6–191.2)	0.036
RSI score on PPIs			
≤13	0/0 (0)		
>13	74/124 (60)	–	–
GERSS score on PPIs			
<18	7/12 (58)		
≥18	67/110 (61)	1.1 (0.3–3.7)	0.862
% total time pH < 4			
≥8 %	4/11 (36)		
<8 %	3/8 (38)	1.1 (0.1–7.1)	0.960
Total DeMeester score			
≥30	1/5 (20)		
<30	4/11 (36)	2.3 (0.2–53.4)	0.519

Data are expressed n/n (%)

*GERD* gastroesophageal reflux disease, *GERD-HRQL* gastroesophageal reflux disease health-related quality of life, *GERSS* gastroesophageal reflux symptom score, *OR* odds ratio, *PPIs* proton-pump inhibitors, *RSI* reflux symptom index

^a^Wald *p* value

The only predictor of a *responsive* outcome of atypical symptoms by univariate analysis was the presence of endoscopic esophagitis (OR = 3.4, CI = 1.4–8.3, *p* = 0.006), Table 6. In these patients, when esophagitis was present preoperatively, a *responsive* outcome was achieved in 82 % (75/92) of patients. If preoperative esophagitis was not present, 44 % (16/32) has a *responsive* outcome.

**Table 6:** Univariate regression analyses for successful or responsive resolution of atypical GERD symptoms

Variable	Successful or responsive outcome	Unadjusted OR (95 % CI)	*p* value^a^
Age (years)			
<50	26/36 (72)		
≥50	67/88 (76)	1.2 (0.5–2.9)	0.648
Gender			
Male	20/30 (67)		
Female	73/94 (78)	1.7 (0.7–4.2)	0.229
Body mass index (kg/m^2^)			
<30	67/92 (73)		
≥30	26/32 (81)	1.6 (0.6–4.8)	0.346
Hiatal hernia			
No	22/35 (63)		
Yes	71/89 (80)	2.3 (1.0–5.5)	0.535
Esophagitis			
No	18/32 (56)		
Yes	75/92 (82)	3.4 (1.4–8.3)	0.006
GERD duration			
<5	17/27 (63)		
≥5	52/87 (60)	0.9 (0.3–2.1)	0.767
PPI duration			
<5	28/42 (67)		
≥5	41/71 (58)	0.7 (0.3–1.5)	0.348
GERD-HRQL score on PPIs			
<15	4/7 (57)		
≥15	89/117 (76)	2.4 (0.4–11.4)	0.274
RSI score on PPIs			
≤13	0/0 (0)		
>13	93/124 (75)	–	–
GERSS score on PPIs			
<18	8/12 (67)		
≥18	85/110 (77)	1.7 (0.4–5.9)	0.417
% total time pH < 4			
≥8 %	6/11 (55)		
<8 %	5/8 (63)	1.4 (0.2–9.7)	0.729
Total DeMeester score			
≥30	2/5 (40)		
<30	6/11 (55)	1.8 (0.2–18.3)	0.592

Data are expressed n/n (%)

*GERD* gastroesophageal reflux disease, *GERD-HRQL* gastroesophageal reflux disease health-related quality of life, *GERSS* gastroesophageal reflux symptom score, *OR* odds ratio, *PPIs* proton-pump inhibitors, *RSI* reflux symptom index

^a^Wald *p* value

Neither duration of disease process nor duration of PPI therapy was found to be predictive of symptomatic outcomes.

### Complications

Two procedures were aborted due to small esophageal tear in the distal esophagus. In both cases, 3 small hemostatic clips around the area of the esophageal tear were successfully used to stop a bleeding. Both patients had no clinical sequelae. All other TF procedure was completed successfully. 13 % (21/156) of patients required hospitalization for more than 24 h due to nausea, anxiety, or post-operative pain. Two patients stayed in the hospital longer than 3 days for pulmonary issues, not related to the TF procedure. 42 % (67/156) of patients reported some post-operative pain at discharge. Of these 67 patients, 4 % (3/67) rated the post-operative pain as severe.

## Discussion

Potent acid-suppressive therapy with PPIs heals many patients with reflux-induced esophageal injury. However, the effectiveness of medical therapy in adequately alleviating reflux symptoms in GERD patients has been more and more questioned, and many patients are dissatisfied with their QOL despite medical therapy. Although patients with a significant response to PPIs and typical GERD symptoms have a high likelihood of responding well to antireflux procedures [5], in truth most of these patients are content to stay on their medication. It is instead patients with an inadequate response to medical therapy (including those with minimal or no response to acid-suppressive therapy) who present for consideration of an antireflux procedure. These patients frequently switch from one PPI to another with little improvement in their QOL [2, 20] and require further work-up (multiple physician and endoscopy suite visits) [2]. This important group of patients has largely been ignored in studies of antireflux surgery, especially in the community settings. Based on our results, it appears that TF may offer those patients (who failed medical treatment and who are generally not good candidates for antireflux surgery [2]) solid symptomatic control without risks associated with traditional surgical option. In cases where TF patients experience recurrent reflux symptoms, most can achieve symptom control with cost effective over-the-counter acid-suppressive medication, as needed.

The current study evaluated predictors of successful symptomatic outcomes of one particular antireflux procedure (TF) in patients with inadequate improvement in symptoms despite PPI therapy, an abnormal QOL on PPI therapy, and objective evidence of GERD. The procedure was performed in 14 community settings using the same technology (EsophyX) by surgeons experienced in and adhering to a similar technique. At the time of study initiation, each participating investigator had performed more than 20 TF procedures. Outcomes were measured clinically using three validated QOL questionnaires, one of which evaluates typical GERD symptoms (GERD-HRQL), one of which evaluates laryngopharyngeal (atypical) symptoms (RSI), and one of which addresses typical and atypical symptoms (GERSS). Success was defined by a reduction in QOL scores and reduction or elimination of PPI therapy. For the purpose of this study, GERSS questionnaire was not used in defining clinical outcomes because it is not specific for typical or atypical presentation of GERD (it is composed of 2 items used to assess typical symptoms and 3 items used to evaluate atypical symptoms).

All patients in the study suffered from troublesome classic GERD symptoms. Multivariate analysis revealed that an elevated GERD-HRQL score (≥15 on PPIs) and ages (≥50 years) were associated with successful outcomes (≥50 % reduction in GERD-HRQL scores and off PPIs). Age ≥ 50 emerged as significant predictor of successful or responsive outcomes. As <30 % of patients in the study were in this age group, it is unclear whether this statistically significant result is of clinical importance.

Patients with typical symptoms responsive to PPI therapy can expect a good response from LF [5]. In this study we found that patients with typical symptoms that persisted at a high level despite PPI therapy (≥15 on GERD-HRQL), and objective evidence of GERD (by endoscopy or reflux testing) could expect a good response to TF. In patients with typical symptoms, two factors associated with successful outcomes on univariate analysis (abnormal RSI score and abnormal GERSS score) did not emerge as significant predictive factors on multivariate analysis. The GERSS questionnaire covers a mixture of atypical and typical symptoms while RSI questionnaire also has a heartburn component. These questionnaires would likely have less weight in a multivariate analysis than the GERD-HRQL which is limited to typical symptoms.

Unlike with successful and responsive outcomes, our study failed to determine significant predictors of poor outcomes. As reported in the previous report from the registry study [6], our population consisted of patients with more severe symptoms and symptoms that persisted despite PPI therapy. Our analysis of 10 patients who underwent revisional procedure showed that 60 % of patients had high preoperative GERD-HRQL (>30) and heartburn score (>20) on a long-term PPI therapy. The complete cohort of the present study, similar to other TF studies [20], may represent a patient population skewed to those with more severe and medically-unresponsive symptoms. Studies of TF in patients with milder and more medically-responsive GERD symptoms may help identify the best patient population for TF [21].

Limitations of the present study include the following factors: (1) QOL assessment preoperatively was routinely performed only with the patient on acid-suppressive medication. Therefore, the degree of response to PPIs as a possible predictive factor could not be assessed; (2) Although all patients had objective evidence of GERD preoperatively, no single diagnostic criterion had to be met for entry. Some patients had esophagitis and no ambulatory reflux testing, others had no esophagitis but abnormal reflux testing. This limited assessment of objective criteria that might be predictive of treatment outcomes; (3) Outcomes were measured clinically with only limited objective follow-up. Although clinical outcomes are the most important, there can be confounding factors and placebo effects that limit the reliability of patients’ self-reported condition. However, our study with almost 2 year follow-up likely negates placebo-effect. Objective testing would be helpful in deciphering these confounding factors. Currently, there are two randomized clinical trials underway with a comprehensive pre- and post-operative testing that should provide additional insights on factors associated with objective outcomes of TF.

Recognizing these limitations, the current study still provides useful information on proper patient selection for the TF procedure. Previous studies reported positive association between lower Hill grade and better outcomes after TF [11, 12]. In this study, pre-operative Hill grade was not positively associated with better outcomes. We believe that this is due to the fact that, based on these earlier studies, we excluded patients with Hill grade IV. We agree with these earlier studies that TF is not appropriate for patients with severe anatomic degradation.

In this study we defined an outcome as responsive, if patients had a good clinical response even if they continued taking PPIs (i.e., with ≥50 % improvement in GERD-HRQL score or normalization in RSI scores regardless of need for ongoing medical therapy, as long as medication use did not increase). We think this was appropriate as our patient population was inadequately controlled by PPIs, and a good therapeutic outcome can be imputed if a patient uncontrolled medically is rendered controlled medically by a therapeutic intervention. Since complete cessation of PPI therapy has been defined as “successful” by research-driven studies, we chose the term “responsive” to indicate a definable clinical outcome. We believe that more research should be devoted to evaluating patients with medically-unresponsive GERD, and that the battle between medical and surgical therapies should instead move toward multimodality management that looks for any combination of therapies that provides the best outcomes for patients. If medically refractory symptoms are controlled by an antireflux procedure, the continued use of medication should not be construed as a failure of anti-reflux surgery.

## Conclusions

This study evaluated preoperative predictors of success in 158 patients with persistent troublesome GERD symptoms despite medical therapy and who underwent an endoluminal therapy (TF). When positive predictors were present, a successful outcome (≥50 % reduction in GERD-HRQL score and no PPI use) was seen in 67 % of patients, and the presence of an elevated GERD-HRQL (≥15) on PPIs before procedure was the best predictor of that outcome. As important if not more, given the failure of medication alone to control symptoms, 84 % of patients normalized their QOL regardless of ongoing medical therapy. In this group, persistence of typical symptoms on medication (initial GERD-HRQL score ≥ 15 on PPIs) and an objectively confirmed diagnosis of GERD (presence of esophagitis) were the best predictors of success. Though further studies will continue to define the role of TF in the treatment of GERD, patients with the characteristics identified herein who have persistent symptoms on PPI therapy can be offered TF with a high likelihood of having a greatly improved QOL afterward.
